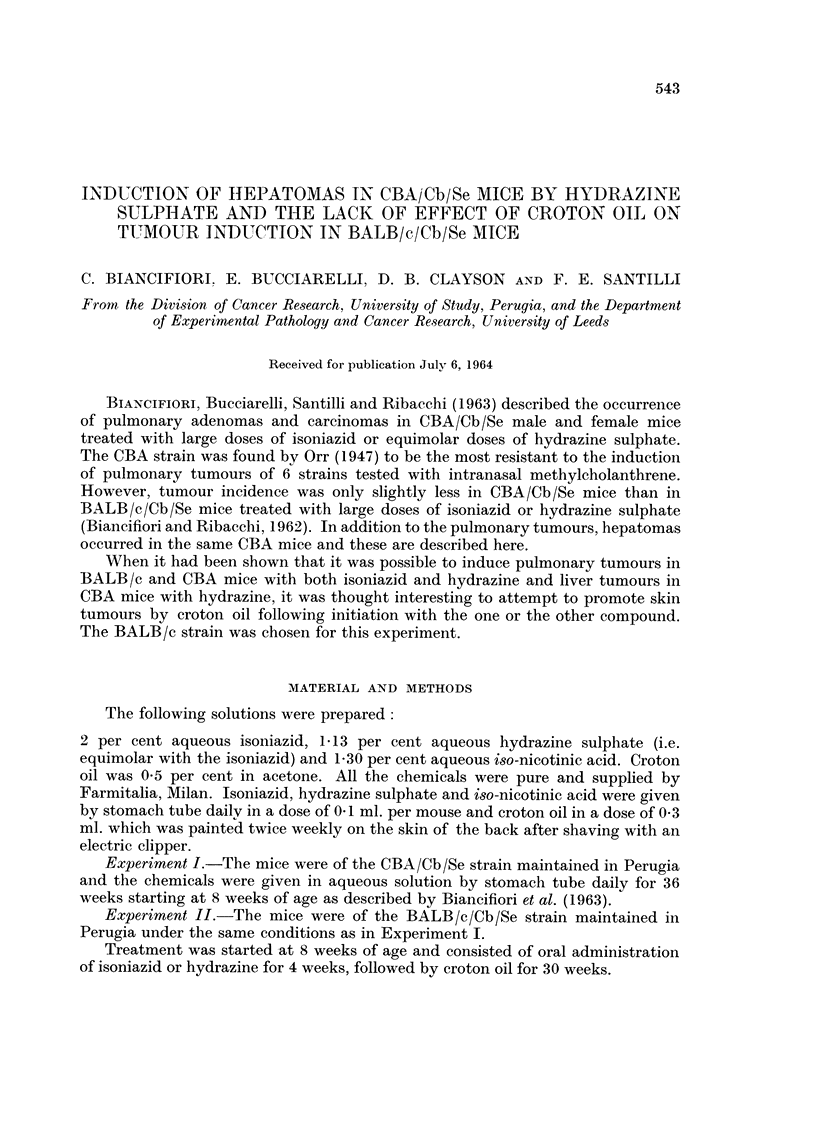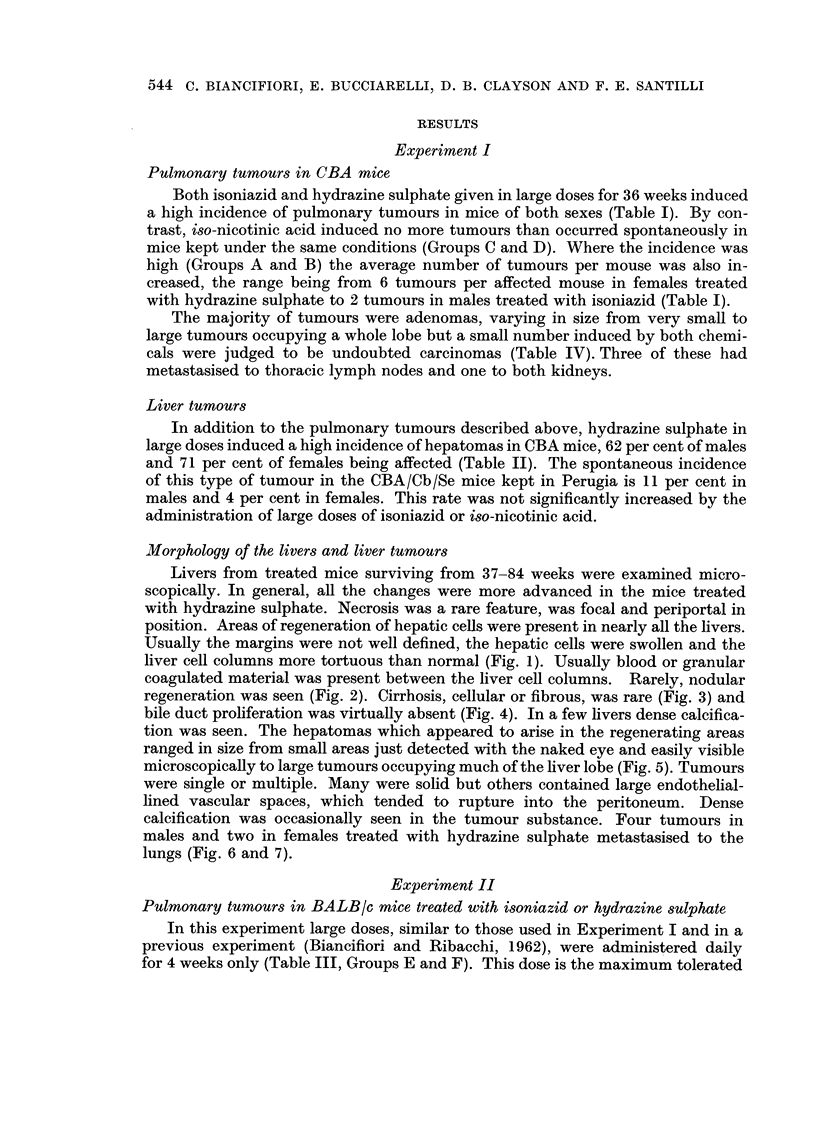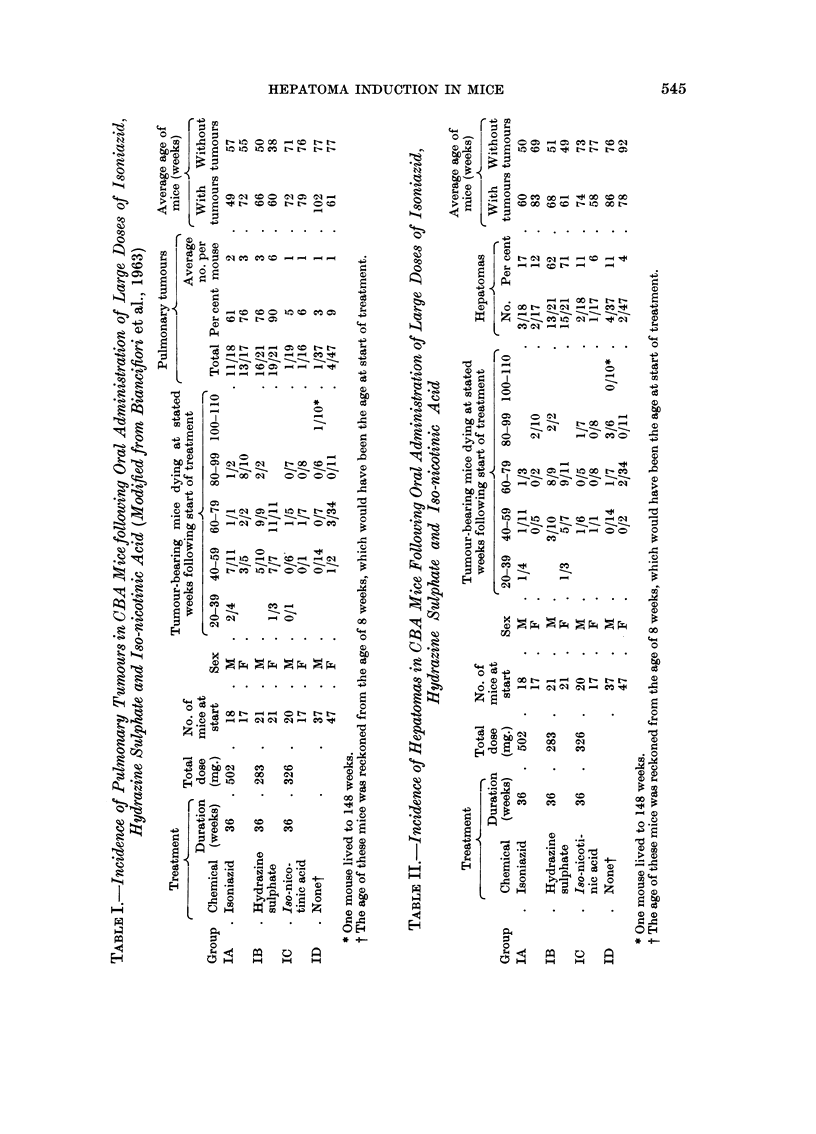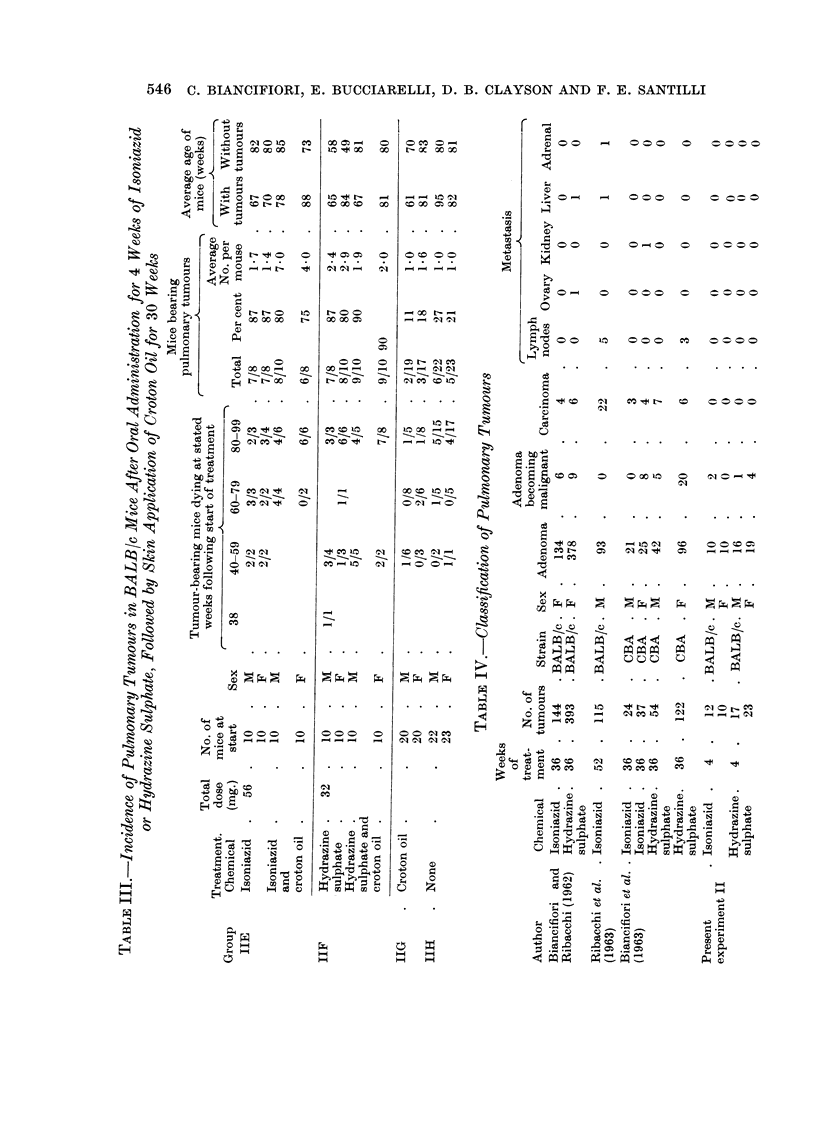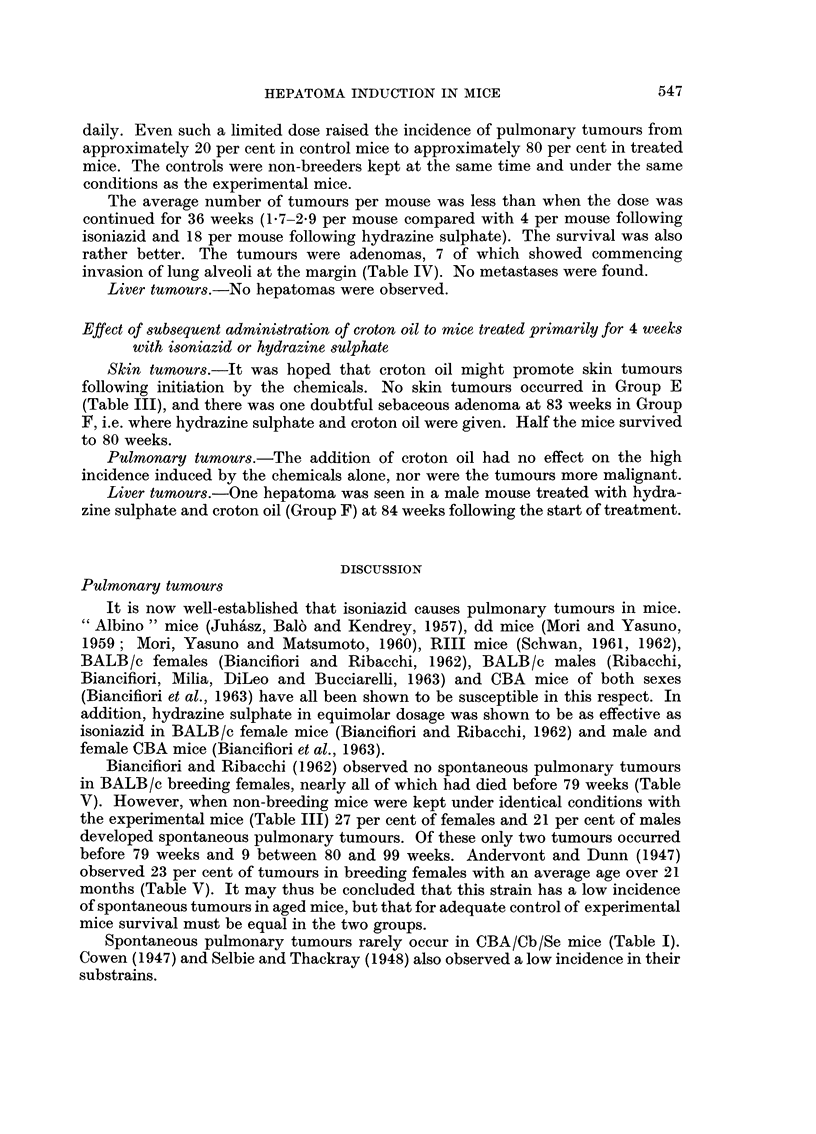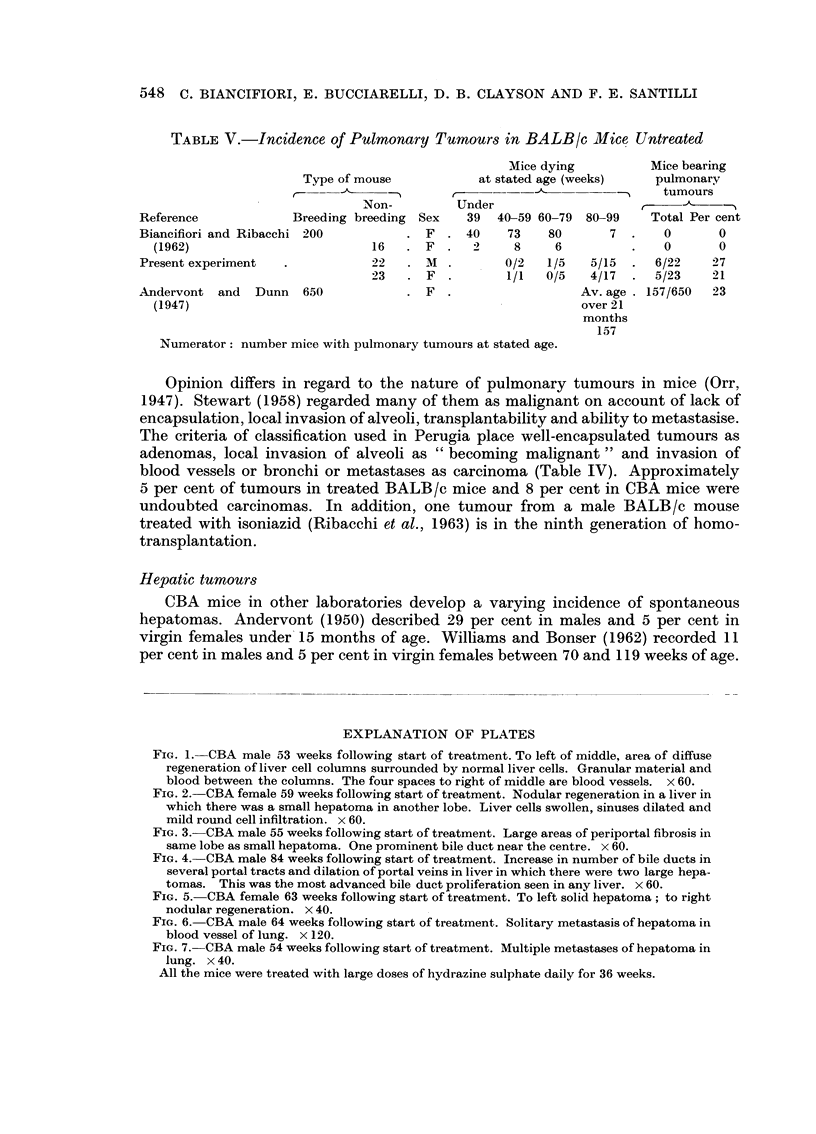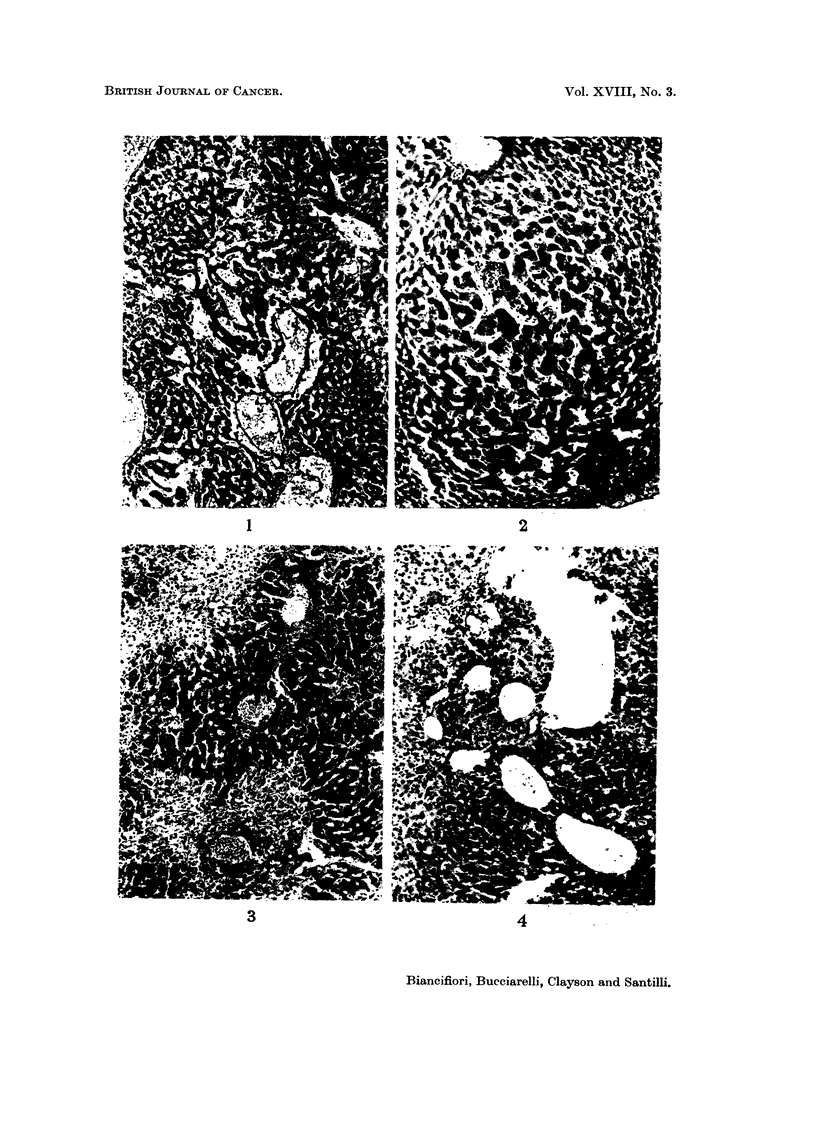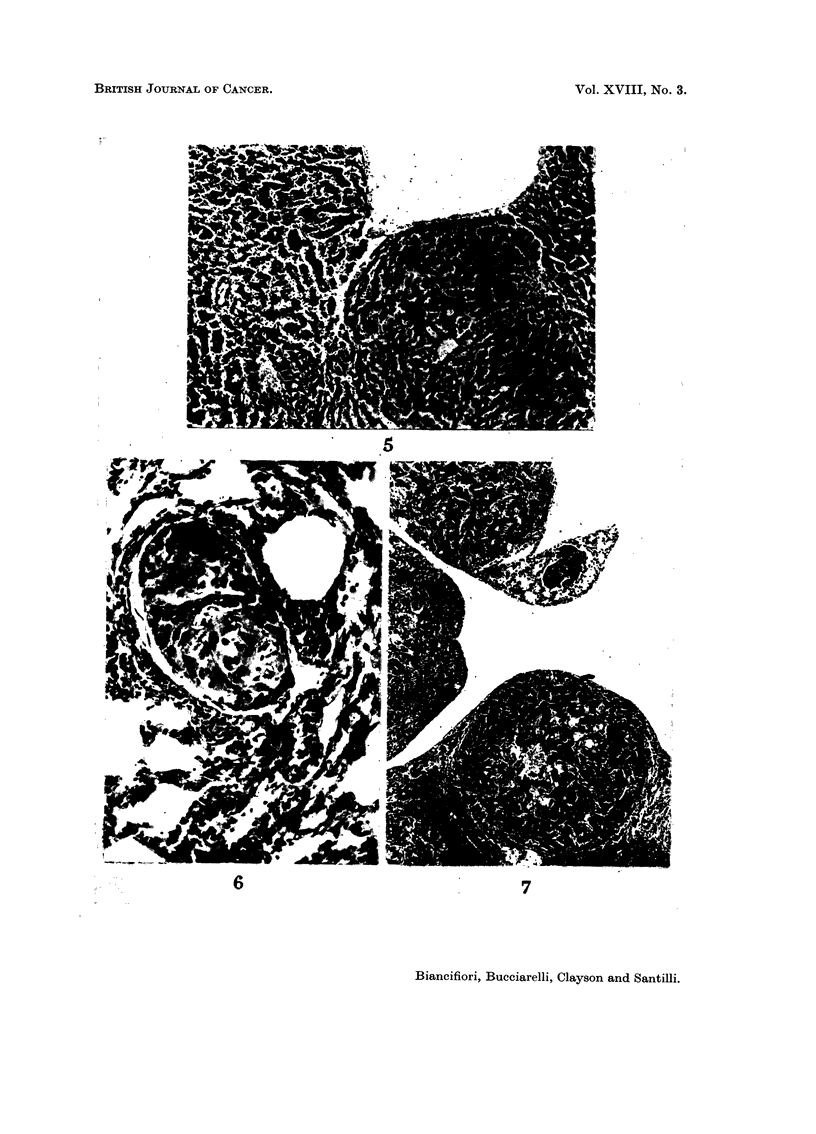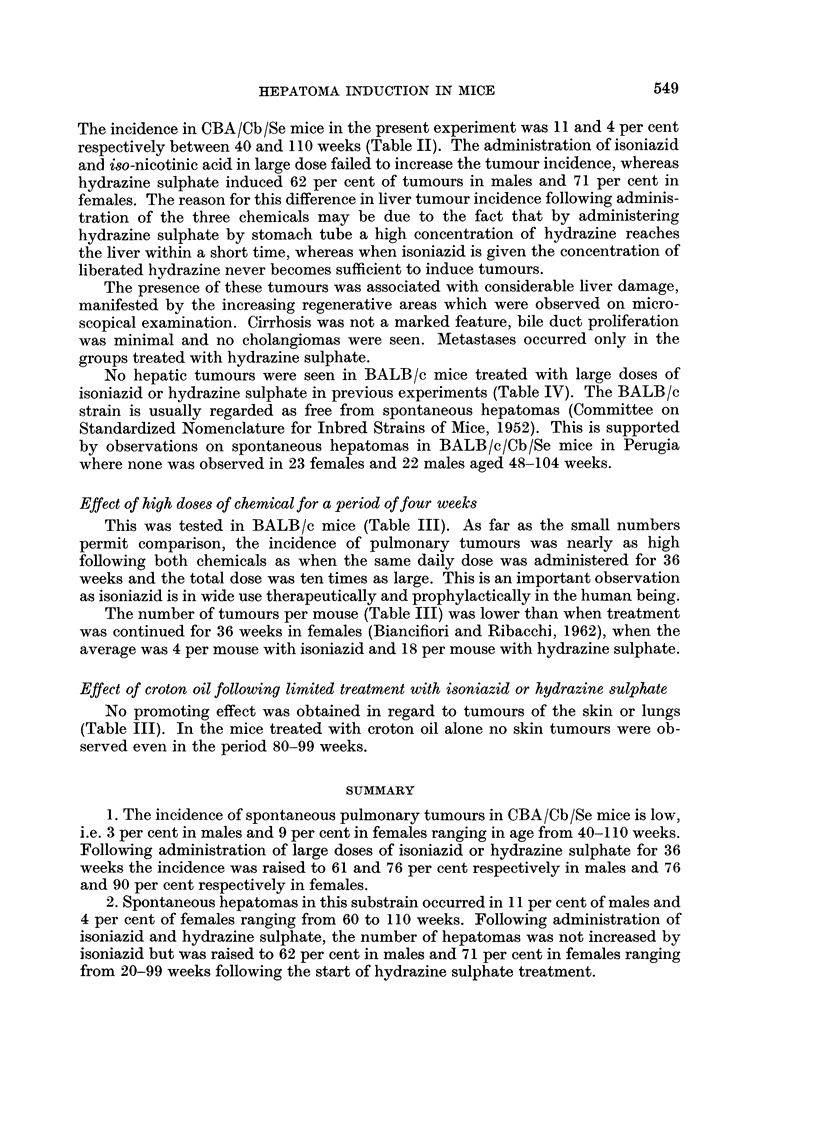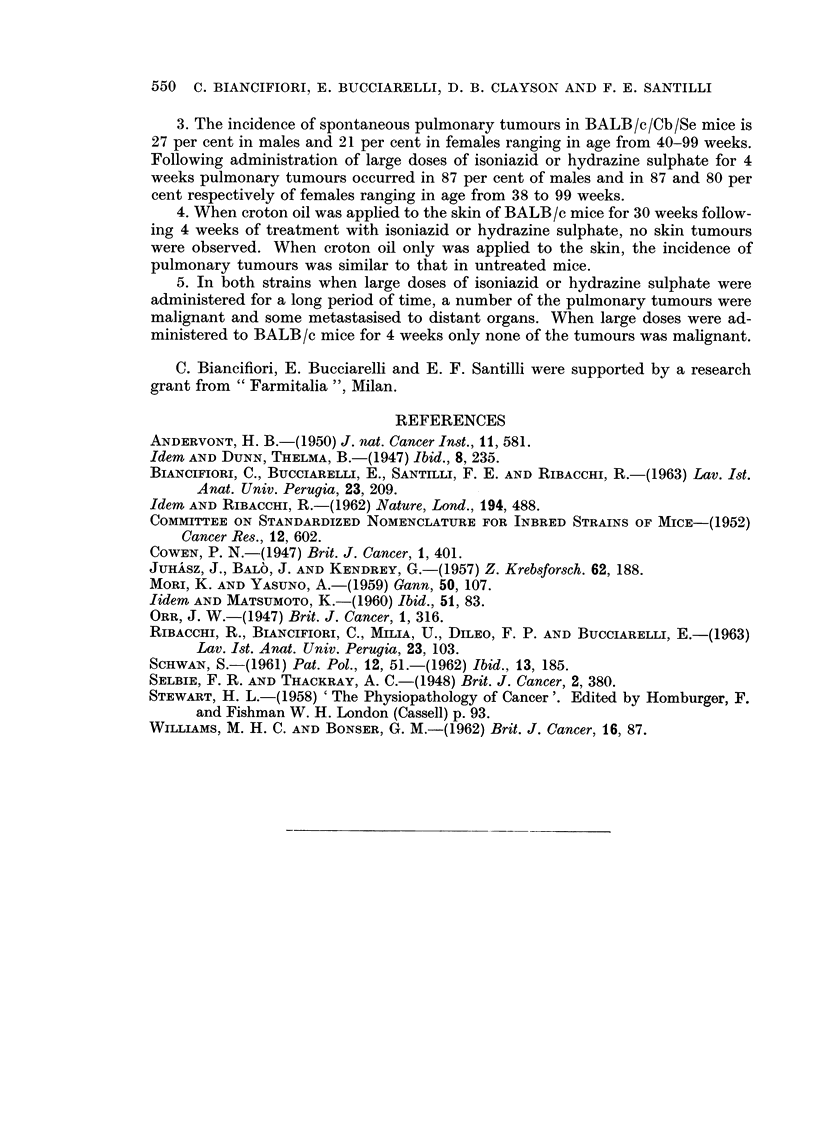# Induction of Hepatomas in CBA/Cb/Se Mice by Hydrazine Sulphate and the Lack of Effect of Croton Oil on Tumour Induction in BALB/c/Cb/Se Mice

**DOI:** 10.1038/bjc.1964.62

**Published:** 1964-09

**Authors:** C. Biancifiori, E. Bucciarelli, D. B. Clayson, F. E. Santilli

## Abstract

**Images:**


### INDUCTION OF HEPATOMAS IN CBA/Cb/Se MICE BY HYDRAZINE SULPHATE AND THE LACK OF EFFECT OF CROTON OIL ON TUMOUR INDUCTION IN BALB/c/Cb/Se MICE

C. BIANCIFIORI, E. BUCCIARELLI, D. B. CLAYSON AND F. E. SANTILLI

*From the Division of Cancer Research, University of Study, Perugia, and the Department of Experimental Pathology and Cancer Research, University of Leeds*

Received for publication July 6, 1964

BIANCIFIORI, Bucciarelli, Santilli and Ribacchi (1963) described the occurrence of pulmonary adenomas and carcinomas in CBA/Cb/Se male and female mice treated with large doses of isoniazid or equimolar doses of hydrazine sulphate. The CBA strain was found by Orr (1947) to be the most resistant to the induction of pulmonary tumours of 6 strains tested with intranasal methylcholanthrene. However, tumour incidence was only slightly less in CBA/Cb/Se mice than in BALB/c/Cb/Se mice treated with large doses of isoniazid or hydrazine sulphate (Biancifiori and Ribacchi, 1962). In addition to the pulmonary tumours, hepatomas occurred in the same CBA mice and these are described here.

When it had been shown that it was possible to induce pulmonary tumours in BALB/c and CBA mice with both isoniazid and hydrazine and liver tumours in CBA mice with hydrazine, it was thought interesting to attempt to promote skin tumours by croton oil following initiation with the one or the other compound. The BALB/c strain was chosen for this experiment.

### MATERIAL AND METHODS

The following solutions were prepared :

2 per cent aqueous isoniazid, 1·13 per cent aqueous hydrazine sulphate (i.e. equimolar with the isoniazid) and 1·30 per cent aqueous *iso*-nicotinic acid. Croton oil was 0·5 per cent in acetone. All the chemicals were pure and supplied by Farmitalia, Milan. Isoniazid, hydrazine sulphate and *iso*-nicotinic acid were given by stomach tube daily in a dose of 0·1 ml. per mouse and croton oil in a dose of 0·3 ml. which was painted twice weekly on the skin of the back after shaving with an electric clipper.

*Experiment I.*—The mice were of the CBA/Cb/Se strain maintained in Perugia and the chemicals were given in aqueous solution by stomach tube daily for 36 weeks starting at 8 weeks of age as described by Biancifiori *et al.* (1963).

*Experiment II.*—The mice were of the BALB/c/Cb/Se strain maintained in Perugia under the same conditions as in Experiment I.

Treatment was started at 8 weeks of age and consisted of oral administration of isoniazid or hydrazine for 4 weeks, followed by croton oil for 30 weeks.

### RESULTS

### *Experiment I*

*Pulmonary tumours in CBA mice*

Both isoniazid and hydrazine sulphate given in large doses for 36 weeks induced a high incidence of pulmonary tumours in mice of both sexes (Table I). By contrast, *iso*-nicotinic acid induced no more tumours than occurred spontaneously in mice kept under the same conditions (Groups C and D). Where the incidence was high (Groups A and B) the average number of tumours per mouse was also increased, the range being from 6 tumours per affected mouse in females treated with hydrazine sulphate to 2 tumours in males treated with isoniazid (Table I).

The majority of tumours were adenomas, varying in size from very small to large tumours occupying a whole lobe but a small number induced by both chemicals were judged to be undoubted carcinomas (Table IV). Three of these had metastasised to thoracic lymph nodes and one to both kidneys.

*Liver tumours*

In addition to the pulmonary tumours described above, hydrazine sulphate in large doses induced a high incidence of hepatomas in CBA mice, 62 per cent of males and 71 per cent of females being affected (Table II). The spontaneous incidence of this type of tumour in the CBA/Cb/Se mice kept in Perugia is 11 per cent in males and 4 per cent in females. This rate was not significantly increased by the administration of large doses of isoniazid or *iso*-nicotinic acid.

*Morphology of the livers and liver tumours*

Livers from treated mice surviving from 37–84 weeks were examined microscopically. In general, all the changes were more advanced in the mice treated with hydrazine sulphate. Necrosis was a rare feature, was focal and periportal in position. Areas of regeneration of hepatic cells were present in nearly all the livers. Usually the margins were not well defined, the hepatic cells were swollen and the liver cell columns more tortuous than normal (Fig. 1). Usually blood or granular coagulated material was present between the liver cell columns. Rarely, nodular regeneration was seen (Fig. 2). Cirrhosis, cellular or fibrous, was rare (Fig. 3) and bile duct proliferation was virtually absent (Fig. 4). In a few livers dense calcification was seen. The hepatomas which appeared to arise in the regenerating areas ranged in size from small areas just detected with the naked eye and easily visible microscopically to large tumours occupying much of the liver lobe (Fig. 5). Tumours were single or multiple. Many were solid but others contained large endothelial-lined vascular spaces, which tended to rupture into the peritoneum. Dense calcification was occasionally seen in the tumour substance. Four tumours in males and two in females treated with hydrazine sulphate metastasised to the lungs (Fig. 6 and 7).

### *Experiment II*

*Pulmonary tumours in BALB/c mice treated with isoniazid or hydrazine sulphate*

In this experiment large doses, similar to those used in Experiment I and in a previous experiment (Biancifiori and Ribacchi, 1962), were administered daily for 4 weeks only (Table III, Groups E and F). This dose is the maximum tolerated daily. Even such a limited dose raised the incidence of pulmonary tumours from approximately 20 per cent in control mice to approximately 80 per cent in treated mice. The controls were non-breeders kept at the same time and under the same conditions as the experimental mice.

The average number of tumours per mouse was less than when the dose was continued for 36 weeks (1·7–2·9 per mouse compared with 4 per mouse following isoniazid and 18 per mouse following hydrazine sulphate). The survival was also rather better. The tumours were adenomas, 7 of which showed commencing invasion of lung alveoli at the margin (Table IV). No metastases were found.

*Liver tumours.*—No hepatomas were observed.

*Effect of subsequent administration of croton oil to mice treated primarily for 4 weeks with isoniazid or hydrazine sulphate*

*Skin tumours.*—It was hoped that croton oil might promote skin tumours following initiation by the chemicals. No skin tumours occurred in Group E (Table III), and there was one doubtful sebaceous adenoma at 83 weeks in Group F, i.e. where hydrazine sulphate and croton oil were given. Half the mice survived to 80 weeks.

*Pulmonary tumours.*—The addition of croton oil had no effect on the high incidence induced by the chemicals alone, nor were the tumours more malignant.

*Liver tumours.*—One hepatoma was seen in a male mouse treated with hydrazine sulphate and croton oil (Group F) at 84 weeks following the start of treatment.

### DISCUSSION

*Pulmonary tumours*

It is now well-established that isoniazid causes pulmonary tumours in mice. " Albino " mice (Juhász, Balò and Kendrey, 1957), dd mice (Mori and Yasuno, 1959; Mori, Yasuno and Matsumoto, 1960), RIII mice (Schwan, 1961, 1962), BALB/c females (Biancifiori and Ribacchi, 1962), BALB/c males (Ribacchi, Biancifiori, Milia, DiLeo and Bucciarelli, 1963) and CBA mice of both sexes (Biancifiori *et al.*, 1963) have all been shown to be susceptible in this respect. In addition, hydrazine sulphate in equimolar dosage was shown to be as effective as isoniazid in BALB/c female mice (Biancifiori and Ribacchi, 1962) and male and female CBA mice (Biancifiori *et al.*, 1963).

Biancifiori and Ribacchi (1962) observed no spontaneous pulmonary tumours in BALB/c breeding females, nearly all of which had died before 79 weeks (Table V). However, when non-breeding mice were kept under identical conditions with the experimental mice (Table III) 27 per cent of females and 21 per cent of males developed spontaneous pulmonary tumours. Of these only two tumours occurred before 79 weeks and 9 between 80 and 99 weeks. Andervont and Dunn (1947) observed 23 per cent of tumours in breeding females with an average age over 21 months (Table V). It may thus be concluded that this strain has a low incidence of spontaneous tumours in aged mice, but that for adequate control of experimental mice survival must be equal in the two groups.

Spontaneous pulmonary tumours rarely occur in CBA/Cb/Se mice (Table I). Cowen (1947) and Selbie and Thackray (1948) also observed a low incidence in their substrains.

TABLE V.—*Incidence of Pulmonary Tumours in BALB/c Mice Untreated*

| Reference | Type of mouse | | Sex | Mice dying at stated age (weeks) | | | | Mice bearing pulmonary tumours | |
|---|---|---|---|---|---|---|---|---|---|
| | Breeding | Non-breeding | | Under 39 | 40–59 | 60–79 | 80–99 | Total | Per cent |
| Biancifiori and Ribacchi | 200 | | F | 40 | 73 | 80 | 7 | 0 | 0 |
| (1962) | | 16 | F | 2 | 8 | 6 | | 0 | 0 |
| Present experiment | | 22 | M | | 0/2 | 1/5 | 5/15 | 6/22 | 27 |
| | | 23 | F | | 1/1 | 0/5 | 4/17 | 5/23 | 21 |
| Andervont and Dunn (1947) | 650 | | F | | | | Av. age over 21 months 157 | 157/650 | 23 |

Numerator : number mice with pulmonary tumours at stated age.

Opinion differs in regard to the nature of pulmonary tumours in mice (Orr, 1947). Stewart (1958) regarded many of them as malignant on account of lack of encapsulation, local invasion of alveoli, transplantability and ability to metastasise. The criteria of classification used in Perugia place well-encapsulated tumours as adenomas, local invasion of alveoli as " becoming malignant " and invasion of blood vessels or bronchi or metastases as carcinoma (Table IV). Approximately 5 per cent of tumours in treated BALB/c mice and 8 per cent in CBA mice were undoubted carcinomas. In addition, one tumour from a male BALB/c mouse treated with isoniazid (Ribacchi *et al.*, 1963) is in the ninth generation of homotransplantation.

*Hepatic tumours*

CBA mice in other laboratories develop a varying incidence of spontaneous hepatomas. Andervont (1950) described 29 per cent in males and 5 per cent in virgin females under 15 months of age. Williams and Bonser (1962) recorded 11 per cent in males and 5 per cent in virgin females between 70 and 119 weeks of age.

---

EXPLANATION OF PLATES

FIG. 1.—CBA male 53 weeks following start of treatment. To left of middle, area of diffuse regeneration of liver cell columns surrounded by normal liver cells. Granular material and blood between the columns. The four spaces to right of middle are blood vessels. ×60.

FIG. 2.—CBA female 59 weeks following start of treatment. Nodular regeneration in a liver in which there was a small hepatoma in another lobe. Liver cells swollen, sinuses dilated and mild round cell infiltration. ×60.

FIG. 3.—CBA male 55 weeks following start of treatment. Large areas of periportal fibrosis in same lobe as small hepatoma. One prominent bile duct near the centre. ×60.

FIG. 4.—CBA male 84 weeks following start of treatment. Increase in number of bile ducts in several portal tracts and dilation of portal veins in liver in which there were two large hepatomas. This was the most advanced bile duct proliferation seen in any liver. ×60.

FIG. 5.—CBA female 63 weeks following start of treatment. To left solid hepatoma ; to right nodular regeneration. ×40.

FIG. 6.—CBA male 64 weeks following start of treatment. Solitary metastasis of hepatoma in blood vessel of lung. ×120.

FIG. 7.—CBA male 54 weeks following start of treatment. Multiple metastases of hepatoma in lung. ×40.

All the mice were treated with large doses of hydrazine sulphate daily for 36 weeks.

1

2

3

4

Biancifiori, Bucciarelli, Clayson and Santilli.

5

6

7

Biancifiori, Bucciarelli, Clayson and Santilli.

The incidence in CBA/Cb/Se mice in the present experiment was 11 and 4 per cent respectively between 40 and 110 weeks (Table II). The administration of isoniazid and *iso*-nicotinic acid in large dose failed to increase the tumour incidence, whereas hydrazine sulphate induced 62 per cent of tumours in males and 71 per cent in females. The reason for this difference in liver tumour incidence following administration of the three chemicals may be due to the fact that by administering hydrazine sulphate by stomach tube a high concentration of hydrazine reaches the liver within a short time, whereas when isoniazid is given the concentration of liberated hydrazine never becomes sufficient to induce tumours.

The presence of these tumours was associated with considerable liver damage, manifested by the increasing regenerative areas which were observed on microscopical examination. Cirrhosis was not a marked feature, bile duct proliferation was minimal and no cholangiomas were seen. Metastases occurred only in the groups treated with hydrazine sulphate.

No hepatic tumours were seen in BALB/c mice treated with large doses of isoniazid or hydrazine sulphate in previous experiments (Table IV). The BALB/c strain is usually regarded as free from spontaneous hepatomas (Committee on Standardized Nomenclature for Inbred Strains of Mice, 1952). This is supported by observations on spontaneous hepatomas in BALB/c/Cb/Se mice in Perugia where none was observed in 23 females and 22 males aged 48–104 weeks.

*Effect of high doses of chemical for a period of four weeks*

This was tested in BALB/c mice (Table III). As far as the small numbers permit comparison, the incidence of pulmonary tumours was nearly as high following both chemicals as when the same daily dose was administered for 36 weeks and the total dose was ten times as large. This is an important observation as isoniazid is in wide use therapeutically and prophylactically in the human being.

The number of tumours per mouse (Table III) was lower than when treatment was continued for 36 weeks in females (Biancifiori and Ribacchi, 1962), when the average was 4 per mouse with isoniazid and 18 per mouse with hydrazine sulphate.

*Effect of croton oil following limited treatment with isoniazid or hydrazine sulphate*

No promoting effect was obtained in regard to tumours of the skin or lungs (Table III). In the mice treated with croton oil alone no skin tumours were observed even in the period 80–99 weeks.

### SUMMARY

1. The incidence of spontaneous pulmonary tumours in CBA/Cb/Se mice is low, i.e. 3 per cent in males and 9 per cent in females ranging in age from 40–110 weeks. Following administration of large doses of isoniazid or hydrazine sulphate for 36 weeks the incidence was raised to 61 and 76 per cent respectively in males and 76 and 90 per cent respectively in females.

2. Spontaneous hepatomas in this substrain occurred in 11 per cent of males and 4 per cent of females ranging from 60 to 110 weeks. Following administration of isoniazid and hydrazine sulphate, the number of hepatomas was not increased by isoniazid but was raised to 62 per cent in males and 71 per cent in females ranging from 20–99 weeks following the start of hydrazine sulphate treatment.

3. The incidence of spontaneous pulmonary tumours in BALB/c/Cb/Se mice is 27 per cent in males and 21 per cent in females ranging in age from 40–99 weeks. Following administration of large doses of isoniazid or hydrazine sulphate for 4 weeks pulmonary tumours occurred in 87 per cent of males and in 87 and 80 per cent respectively of females ranging in age from 38 to 99 weeks.

4. When croton oil was applied to the skin of BALB/c mice for 30 weeks following 4 weeks of treatment with isoniazid or hydrazine sulphate, no skin tumours were observed. When croton oil only was applied to the skin, the incidence of pulmonary tumours was similar to that in untreated mice.

5. In both strains when large doses of isoniazid or hydrazine sulphate were administered for a long period of time, a number of the pulmonary tumours were malignant and some metastasised to distant organs. When large doses were administered to BALB/c mice for 4 weeks only none of the tumours was malignant.

C. Biancifiori, E. Bucciarelli and E. F. Santilli were supported by a research grant from " Farmitalia ", Milan.

### REFERENCES

ANDERVONT, H. B.—(1950) *J. nat. Cancer Inst.*, **11**, 581.
*Idem* AND DUNN, THELMA, B.—(1947) *Ibid.*, **8**, 235.
BIANCIFIORI, C., BUCCIARELLI, E., SANTILLI, F. E. AND RIBACCHI, R.—(1963) *Lav. Ist. Anat. Univ. Perugia*, **23**, 209.
*Idem* AND RIBACCHI, R.—(1962) *Nature, Lond.*, **194**, 488.
COMMITTEE ON STANDARDIZED NOMENCLATURE FOR INBRED STRAINS OF MICE—(1952) *Cancer Res.*, **12**, 602.
COWEN, P. N.—(1947) *Brit. J. Cancer*, **1**, 401.
JUHÁSZ, J., BALÒ, J. AND KENDREY, G.—(1957) *Z. Krebsforsch.* **62**, 188.
MORI, K. AND YASUNO, A.—(1959) *Gann*, **50**, 107.
*Iidem* AND MATSUMOTO, K.—(1960) *Ibid.*, **51**, 83.
ORR, J. W.—(1947) *Brit. J. Cancer*, **1**, 316.
RIBACCHI, R., BIANCIFIORI, C., MILIA, U., DILEO, F. P. AND BUCCIARELLI, E.—(1963) *Lav. Ist. Anat. Univ. Perugia*, **23**, 103.
SCHWAN, S.—(1961) *Pat. Pol.*, **12**, 51.—(1962) *Ibid.*, **13**, 185.
SELBIE, F. R. AND THACKRAY, A. C.—(1948) *Brit. J. Cancer*, **2**, 380.
STEWART, H. L.—(1958) ' The Physiopathology of Cancer '. Edited by Homburger, F. and Fishman W. H. London (Cassell) p. 93.
WILLIAMS, M. H. C. AND BONSER, G. M.—(1962) *Brit. J. Cancer*, **16**, 87.